# Horsepox: Framing a dual use research of concern debate

**DOI:** 10.1371/journal.ppat.1007344

**Published:** 2018-10-04

**Authors:** Carolyn B. Coyne

**Affiliations:** Department of Pediatrics, University of Pittsburgh School of Medicine, Pittsburgh, Pennsylvania, United States of America

The implications of publishing dual use research of concern (DURC) continues to be a topic widely and deeply discussed in the scientific community. In a series of Opinion articles, *PLOS Pathogens* intends to contribute to this ongoing debate with four balanced and differing perspectives from notable members of the scientific community.

The four author groups represented in these Opinions include Drs. Ryan S. Noyce and David H. Evans, two authors of a recent *PLOS ONE* study on Horsepox virus [1], Dr. Volker Thiel, who served as Academic Editor for the article describing the study, and Dr. Thomas Inglesby, Jr. and Dr. Kevin M. Esvelt, both of whom have advocated for changes in the peer reviewing and publishing of DURC-related research. These authors, using the *PLOS ONE* paper as a springboard for their Opinions, have each provided insightful commentary related to DURC studies and the possible impact of these studies on infectious diseases research more generally.

In early 2018, Ryan S. Noyce, Seth Lederman, and David H. Evans described a *de novo* approach to synthesize the complete sequence of a horsepox virus (HPVX), which has not been detected in the wild since the mid-1980s [1]. The authors aimed to develop strategies to design HPVX-based vaccine approaches to improve existing vaccinia virus (VACV)-based live vaccinations. This study included the highly detailed methods the authors used to synthesize HPVX *de novo* and a discussion of how these methods could be used to develop other live virus-based vaccines. The publishing of this study led to intense debate among the public and scientific community as to whether this type of study should be permissible given that the methods described could be used to synthesize any poxvirus, particularly variola virus, which was declared eradicated in 1980 and is the infectious agent responsible for smallpox.

In light of the debate around the *PLOS ONE* article, the editors of *PLOS Pathogens* felt that providing a forum for individuals holding opinions on both sides of this debate to explain their rationale would be appropriate and interesting, particularly given the broad infectious diseases readership of the journal. As a *PLOS Pathogens* Opinions editor, I was eager to facilitate this process and guide the four papers through peer review and publication. The intent of publishing these Opinions together is to facilitate and encourage a healthy and balanced discussion of the implications of publishing DURC-related research and to provide a forum to consider the HPVX study as a model by which to consider future similar work. Together, and along with the original article in *PLOS ONE*, these four Opinions form a compendium of commentaries on this important topic. I hope that you find these articles as thought-provoking as I did and that they allow you to consider all sides of this debate as you form your own opinions.
